# Augmentation of cognitive brain functions with transcranial lasers

**DOI:** 10.3389/fnsys.2014.00036

**Published:** 2014-03-14

**Authors:** F. Gonzalez-Lima, Douglas W. Barrett

**Affiliations:** Department of Psychology and Institute for Neuroscience, University of Texas at AustinAustin, TX, USA

**Keywords:** cognitive enhancement, cytochrome oxidase, low-level light therapy, brain stimulation, photoneuromodulation

Discovering that transcranial infrared laser stimulation produces beneficial effects on frontal cortex functions such as sustained attention, working memory, and affective state has been groundbreaking. Transcranial laser stimulation with low-power density (mW/cm^2^) and high-energy density (J/cm^2^) monochromatic light in the near-infrared wavelengths modulates brain functions and may produce neurotherapeutic effects in a nondestructive and non-thermal manner (Lampl, 2007; Hashmi et al., 2010). Barrett and Gonzalez-Lima (2013) provided the first controlled study showing that transcranial laser stimulation improves human cognitive and emotional brain functions. But for the field of low-level light/laser therapy (LLLT), development of a model of how luminous energy from red-to-near-infrared wavelengths modulates bioenergetics began with *in vitro* and *in vivo* discoveries in the last 40 years. Previous LLLT reviews have provided extensive background about historical developments, principles and applications (Rojas and Gonzalez-Lima, 2011, 2013; Chung et al., 2012). The purpose of this paper is to provide an update on LLLT's neurochemical mechanisms supporting transcranial laser stimulation for cognitive-enhancing applications. We will explain first LLLT's action on brain bioenergetics, briefly describe its bioavailability and dose-response, and finish with its beneficial effects on cognitive functions. Although our focus is on prefrontal-related cognitive functions, in principle LLLT should be able to modulate other brain functions. For example, stimulating different brain regions should affect different functions related to sensory and motor systems.

## Brain bioenergetics

The way that near-infrared lasers and light-emitting diodes (LEDs) interact with brain function is based on bioenergetics, a mechanism that is fundamentally different than that of other brain stimulation methods such as electric and magnetic stimulation. LLLT has been found to modulate the function of neurons in cell cultures, brain function in animals, and cognitive and emotional functions in healthy persons and clinical conditions. Photoneuromodulation involves the absorption of photons by specific molecules in neurons that activate bioenergetic signaling pathways after exposure to red-to-near-infrared light. The 600–1150 nm wavelengths allow better tissue penetration by photons because light is scattered at lower wavelengths and absorbed by water at higher wavelengths (Hamblin and Demidova, 2006). Over 25 years ago, it was found that molecules that absorb LLLT wavelengths are part of the mitochondrial respiratory enzyme cytochrome oxidase in different oxidation states (Karu et al., 2005). Thus, for red-to-near-infrared light, the primary molecular *photoacceptor* of photon energy is cytochrome oxidase (also called cytochrome *c* oxidase or cytochrome *a-a3*) (Pastore et al., 2000).

Therefore, photon energy absorption by cytochrome oxidase is well-established as the primary neurochemical mechanism of action of LLLT in neurons (Wong-Riley et al., 2005). The more the enzymatic activity of cytochrome oxidase increases, the more metabolic energy that is produced via mitochondrial oxidative phosphorylation. LLLT supplies the brain with metabolic energy in a way analogous to the conversion of nutrients into metabolic energy, but with light instead of nutrients providing the source for ATP-based metabolic energy (Mochizuki-Oda et al., 2002). If an effective near-infrared light energy dose is supplied, it stimulates brain ATP production (Lapchak and De Taboada, 2010) and blood flow (Uozumi et al., 2010), thereby fueling ATP-dependent membrane ion pumps, leading to greater membrane stability and resistance to depolarization, which has been shown to transiently reduce neuronal excitability (Konstantinovic et al., 2013). On the other hand, electromagnetic stimulation directly changes the electrical excitability of neurons.

A long-lasting effect is achieved by LLLT's up-regulating the amount of cytochrome oxidase, which enhances neuronal capacity for metabolic energy production that may be used to support cognitive brain functions. In mice and rats, memory has been improved by LLLT (Michalikova et al., 2008; Rojas et al., 2012a) and by methylene blue, a drug that at low doses donates electrons to cytochrome oxidase (Rojas et al., 2012b). Near-infrared light stimulates mitochondrial respiration by donating photons to cytochrome oxidase, because cytochrome oxidase is the main acceptor of photons from red-to-near-infrared light in neurons. By persistently stimulating cytochrome oxidase activity, transcranial LLLT induces post-stimulation up-regulation of the amount of cytochrome oxidase in brain mitochondria (Rojas et al., 2012a). Therefore, LLLT may lead to the conversion of luminous energy into metabolic energy (during light exposure) and to the up-regulation of the mitochondrial enzymatic machinery to produce more energy (after light exposure).

## Bioavailability and hormetic dose-response

The most abundant metalloprotein in nerve tissue is cytochrome oxidase, and its absorption wavelengths are well correlated with its enzymatic activity and ATP production (Wong-Riley et al., 2005). High LLLT bioavailability to the brain *in vivo* has been shown by inducing brain cytochrome oxidase activity transcranially, leading to enhanced extinction memory retention in normal rats (Rojas et al., 2012a) and improved visual discrimination in rats with impaired retinal mitochondrial function (Rojas et al., 2008). Our LLLT studies utilized varied wavelengths (633–1064 nm), daily doses (1–60 J/cm^2^), fractionation sessions (1–6), and power densities (2–250 mW/cm^2^) that identified effective LLLT parameters for rats and humans.

For example, we tested in rats the effects of different LLLT doses *in vivo* on brain cytochrome oxidase activity, at either 10.9, 21.6, 32.9 J/cm^2^, or no LLLT. Treatments were delivered for 20, 40, and 60 min via four 660-nm LED arrays with a power density of 9 mW/cm^2^. One day after the LLLT session, brains were extracted, frozen, sectioned, and processed for cytochrome oxidase histochemistry. A 10.9 J/cm^2^ dose increased cytochrome oxidase activity by 13.6%. A 21.6 J/cm^2^ dose produced a 10.3% increase. A non-significant cytochrome oxidase increase of 3% was found after the highest 32.9 J/cm^2^ dose. Responses of brain cytochrome oxidase to LLLT *in vivo* were characterized by hormesis, with a low dose being stimulatory, while higher doses were less effective.

The first demonstration that LLLT increased oxygen consumption in the rat prefrontal cortex *in vivo* was provided by Rojas et al. (2012a). Oxygen concentration in the cortex of rats was measured using fluorescence-quenching during LLLT at 9 mW/cm^2^ and 660 nm. LLLT induced a dose-dependent increase in oxygen consumption of 5% after 1 J/cm^2^ and 16% after 5 J/cm^2^. Since oxygen is used to form water within mitochondria in a reaction catalyzed by cytochrome oxidase, more cytochrome oxidase activity should lead to more oxygen consumption.

LLLT may offer some advantages over other types of stimulation, because LLLT non-invasively targets cytochrome oxidase, a key enzyme for energy production, with induced expression linked to energy demand. Hence LLLT is mechanistically specific and non-invasive, while transcranial magnetic stimulation may be non-specific, prolonged forehead electrical stimulation may produce muscle spasms, and deep brain or vagus nerve stimulations are invasive.

## Cognitive and emotional functions

LLLT via commercial low-power sources (such as FDA-cleared laser diodes and LEDs) is a highly promising, affordable, non-pharmacological alternative for improving cognitive function. LLLT delivers safe doses of light energy that are sufficiently high to modulate neuronal functions, but low enough to not result in any damage (Wong-Riley et al., 2005). In 2002, the FDA approved LLLT for pain relief in cases of head and neck pain, arthritis and carpal tunnel syndrome (Fulop et al., 2010). LLLT has been used non-invasively in humans after ischemic stroke to improve neurological outcome (Lampl et al., 2007). It also led to improved recovery and reduced fatigue after exercise (Leal Junior et al., 2010). One LLLT stimulation session to the forehead, as reported by Schiffer et al. (2009), produced a significant antidepressant effect in depressed patients. No adverse side effects were found either immediately or at 2 or 4 weeks after LLLT. Thus, these beneficial LLLT treatments have been found to be safe in humans. Even though LLLT has been regarded as safe and received FDA approval for pain treatment, the use of transcranial lasers for cognitive augmentation should be restricted to research until further controlled studies support this application for clinical use.

We used transcranial laser stimulation to the forehead in a placebo-controlled, randomized study, to influence cognitive tasks related to the prefrontal cortex, including a psychomotor vigilance task (PVT) and a delayed match-to-sample (DMS) memory task (Barrett and Gonzalez-Lima, 2013). The PVT assesses sustained attention, with participants remaining vigilant during delay intervals, and pushing a button when a visual stimulus appears on a monitor. Our laser stimulation targeted prefrontal areas which are implicated in the sustained attentional processes of the PVT (Drummond et al., 2005). Similarly, the DMS task engages the prefrontal cortex as part of a network of frontal and parietal brain regions (Nieder and Miller, 2004).

**Figure 1:**
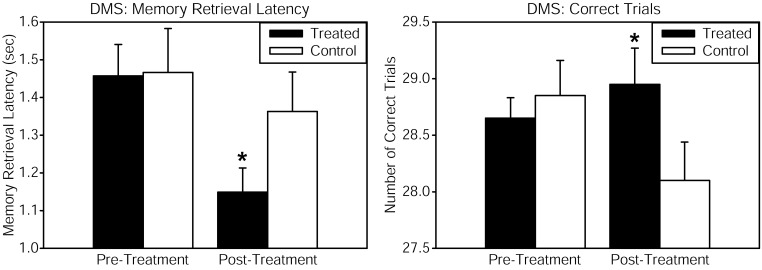
**Cognitive performance in the delayed match-to-sample (DMS) memory task was improved after transcranial infrared stimulation to the right forehead**. The DMS task involves presentation of a visual stimulus (grid pattern) on a screen. Then the stimulus disappears, and the participant must remember the stimulus through a delay. Then two choices appear, and the participant must decide which of these two is identical to the previous stimulus (the “match”). Treated subjects showed faster memory retrieval (left panel) and increased number of correct trials (right panel) out of 30 trials when attempting to choose the correct grid pattern. The function of frontal cortex regions, implicated in the attentional mode network utilized during this visuospatial memory task, was augmented by the laser treatment. Compared to baseline, this treatment also increased by 5% the oxyhemoglobin concentration of the prefrontal cortex as measured by near-infrared spectroscopy, both during the laser stimulation and during post-treatment DMS performance (in preparation). The data for the treated group consisted of *n* = 10 males and *n* = 10 females; the control group also consisted of *n* = 10 males and *n* = 10 females. ^*^Significant treatment by pre-post score interaction, *p* < 0.05.

Healthy volunteers received continuous wave near-infrared light intersecting cytochrome oxidase's absorption spectrum, delivered to the forehead using a 1064 nm low-power laser diode (also known as “cold laser”), which maximizes tissue penetration due to its long wavelength, and has been used in humans for other indications. The power density (or irradiance), 250 mW/cm^2^, as well as the cumulative energy density (or fluence), 60 J/cm^2^, were the same that showed beneficial psychological effects in Schiffer et al. (2009). This laser exposure produces negligible heat and no physical damage at the low power level used. This laser apparatus is used safely in a clinical setting by the supplier of the laser (Cell Gen Therapeutics, HD Laser Center, Dallas, TX). Reaction time in the PVT was improved by the laser treatment, as shown by a significant pre-post reaction time change relative to the placebo group. The DMS memory task also revealed significant enhancements in measures of memory retrieval latency and number of correct trials, when comparing the LLLT-treated with the placebo group (Figure 1). Self-reported positive and negative affective (emotional) states were also measured using the PANAS-X questionnaire before and 2 weeks after laser treatment. As compared to the placebo, treated subjects reported significantly improved affective states. We suggest that this kind of transcranial laser stimulation may serve as a non-invasive and efficacious method to augment cognitive brain functions related to attention, memory, and emotional functions.

LLLT's bioenergetics mechanisms leading to cognitive augmentation may also be at play in its neuroprotective effects (Gonzalez-Lima et al., 2013). LLLT's stimulation of mitochondrial respiration should improve cellular function due to increased metabolic energy, as well as cellular survival after injury, due to the antioxidant effects of increases in cytochrome oxidase and superoxide dismutase (Rojas et al., 2008).

Laser transmittance of the 1064-nm wavelength at the forehead LLLT site was estimated in a post-mortem human specimen, which showed that approximately 2% of the light passed through the frontal bone. This yielded an absorption coefficient of *a* = 0.24, similar to the reported *a* = 0.22 transmittance through cranial bone for this wavelength (Bashkatov and Genina, 2006). Thus, we estimated that about 1.2 J/cm^2^ of the 60 J/cm^2^ LLLT dose applied reached the surface of the prefrontal cortex. This value is similar to 1 J/cm^2^, the peak effective LLLT dose in neuron cultures for increasing cytochrome oxidase activity (Rojas and Gonzalez-Lima, 2011).

## Conclusions

Transcranial absorption of photon energy by cytochrome oxidase, the terminal enzyme in mitochondrial respiration, is proposed as the bioenergetic mechanism of action of LLLT in the brain. Transcranial LLLT up-regulates cortical cytochrome oxidase and enhances oxidative phosphorylation. LLLT improves prefrontal cortex-related cognitive functions, such as sustained attention, extinction memory, working memory, and affective state. Transcranial infrared stimulation may be used efficaciously to support neuronal mitochondrial respiration as a new non-invasive, cognition-improving intervention in animals and humans. This fascinating new approach should also be able to influence other brain functions depending on the neuroanatomical site stimulated and the stimulation parameters used.
